# Generic dynamic causal modelling: An illustrative application to Parkinson's disease

**DOI:** 10.1016/j.neuroimage.2018.08.039

**Published:** 2018-08-18

**Authors:** Bernadette C.M. van Wijk, Hayriye Cagnan, Vladimir Litvak, Andrea A. Kühn, Karl J. Friston

**Affiliations:** a Integrative Model-based Cognitive Neuroscience Research Unit, Department of Psychology, University of Amsterdam, The Netherlands; b Department of Neurology, Charité - University Medicine Berlin, Germany; c Wellcome Centre for Human Neuroimaging, UCL Institute of Neurology, London, UK; d MRC Brain Network Dynamics Unit (BNDU), Department of Pharmacology and Nuffield Department of Clinical Neurosciences, University of Oxford, UK

**Keywords:** Dynamic causal modelling, Neural mass models, Oscillations, Basal ganglia, Motor cortex, Parkinson's disease

## Abstract

We present a technical development in the dynamic causal modelling of electrophysiological responses that combines qualitatively different neural mass models within a single network. This affords the option to couple various cortical and subcortical nodes that differ in their form and dynamics. Moreover, it enables users to implement new neural mass models in a straightforward and standardized way. This generic framework hence supports flexibility and facilitates the exploration of increasingly plausible models. We illustrate this by coupling a basal ganglia-thalamus model to a (previously validated) cortical model developed specifically for motor cortex. The ensuing DCM is used to infer pathways that contribute to the suppression of beta oscillations induced by dopaminergic medication in patients with Parkinson's disease. Experimental recordings were obtained from deep brain stimulation electrodes (implanted in the subthalamic nucleus) and simultaneous magnetoencephalography. In line with previous studies, our results indicate a reduction of synaptic efficacy within the circuit between the subthalamic nucleus and external pallidum, as well as reduced efficacy in connections of the hyperdirect and indirect pathway leading to this circuit. This work forms the foundation for a range of modelling studies of the synaptic mechanisms (and pathophysiology) underlying event-related potentials and cross-spectral densities.

## 1 Introduction

One of the most challenging objectives in neuroscience is to translate experimental observations into neuronal mechanisms. Computational models – using plausible descriptions of neural dynamics – are crucial for this purpose. Dynamic causal modelling (DCM) was originally developed to infer effective connectivity within a distributed network of brain regions generating task-based fMRI responses (Friston et al., 2003). This was followed by an application to EEG/MEG responses (David et al., 2006). Further developments enabled the use of DCM in task-free designs (Moran et al., 2009; Friston et al., 2014). The core of each DCM is a set of differential equations describing neural population responses to endogenous synaptic input, from within the brain, or exogenous stimuli. These equations are combined with an observation function that maps unobserved (i.e., hidden) neural states to data measurements.

The type of information afforded by DCM depends on the generative model used and the spatiotemporal resolution of the imaging modality. For electrophysiological time series in particular, one could (in principle) use a wide range of neural mass (or field) models that vary in their level of biological detail (Deco et al., 2008). Accordingly, the suite of models implemented in DCM has been continuously elaborated over the years (Moran et al., 2013). Models for EEG and MEG have been inspired by the laminar organization of neocortex and include separate populations for spiny stellate cells, inhibitory interneurons, and pyramidal cells for each source in a network (David et al., 2006). Within DCM, most model variations are available in a convolution-based (Jansen and Rit, 1995) and a conductance-based (Morris and Lecar, 1981) form, and have been implemented as neural masses as well as fields (Pinotsis et al., 2012). Furthermore, researchers have used bespoke DCMs that are adaptations of these models (Youssofzadeh et al., 2015; Bhatt et al., 2016; Papadopoulou et al., 2016; Shaw et al., 2017) or have developed subcortical models to address specific research questions (Moran et al., 2011a; Marreiros et al., 2013).

**Fig. 1:**
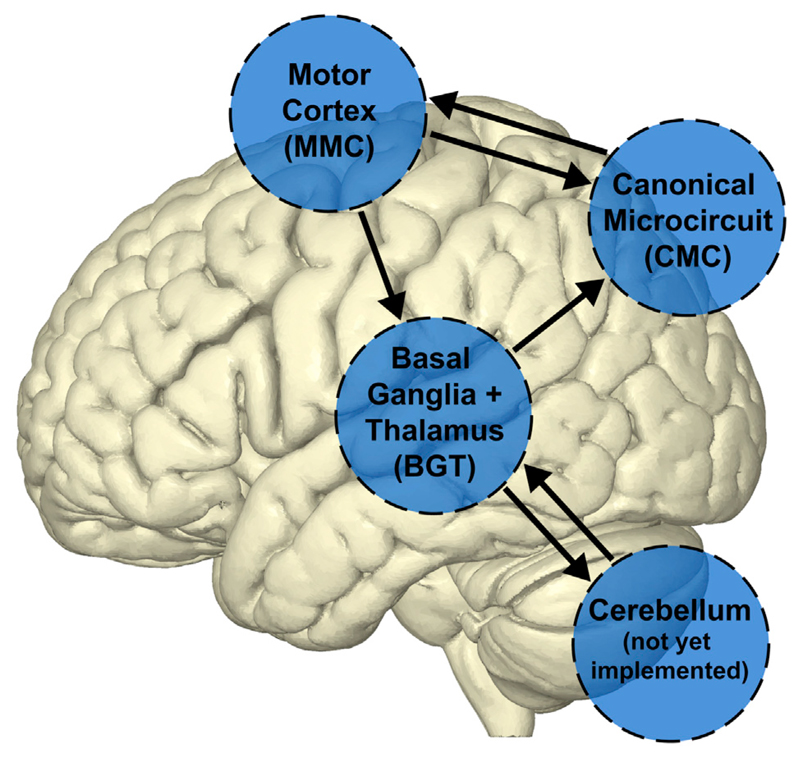
Generic DCM supports different types of neural mass models within the same network. Depicted is a hypothetical network between some of the currently implemented neural mass models ('CMC', 'MMC', 'BGT') and a to-be-constructed model in the cerebellum.

In this paper, we present a generalization in the implementation of DCM that accommodates a combination of different types of neural mass models within a single network (see Fig. 1). This is an important step towards the flexible use of DCM for studies in which individual regions require a distinct dynamical description – due to differences in microcircuitry or laminar organization. This would, for example, apply to a network containing cortical and subcortical regions, and/or the cerebellum. The new generic framework also provides a straightforward way of implementing new models, thereby enabling users to add to a portfolio of models for brain structures that have not yet been studied with DCM. We illustrate this framework using a cortico-basal ganglia-thalamus circuit model to investigate the pathways involved in the suppression of beta oscillations with dopaminergic medication in Parkinson's disease, as seen in simultaneous MEG and LFP recordings. Although our example application takes spectral densities as the to-be-predicted data features, the methods described could also be readily applied to event-related potentials.

## 2 Implementation

We developed generic DCM to finesse a number of restrictions in the standard implementation. Specifically, the aim of this work was three-fold: 1) to allow for coupling between sources that are described by different (versions of) neural models; 2) to enable users to implement new neural models and integrate them within the DCM framework; 3) to give users full control over the specification of condition-specific effects on intrinsic synaptic parameters. We note that the standard DCM implementation is still available in unchanged form and is computationally optimized for networks, where each source is described with the same type of model.

### 2.1 Standard DCM implementation

**Fig. 2:**
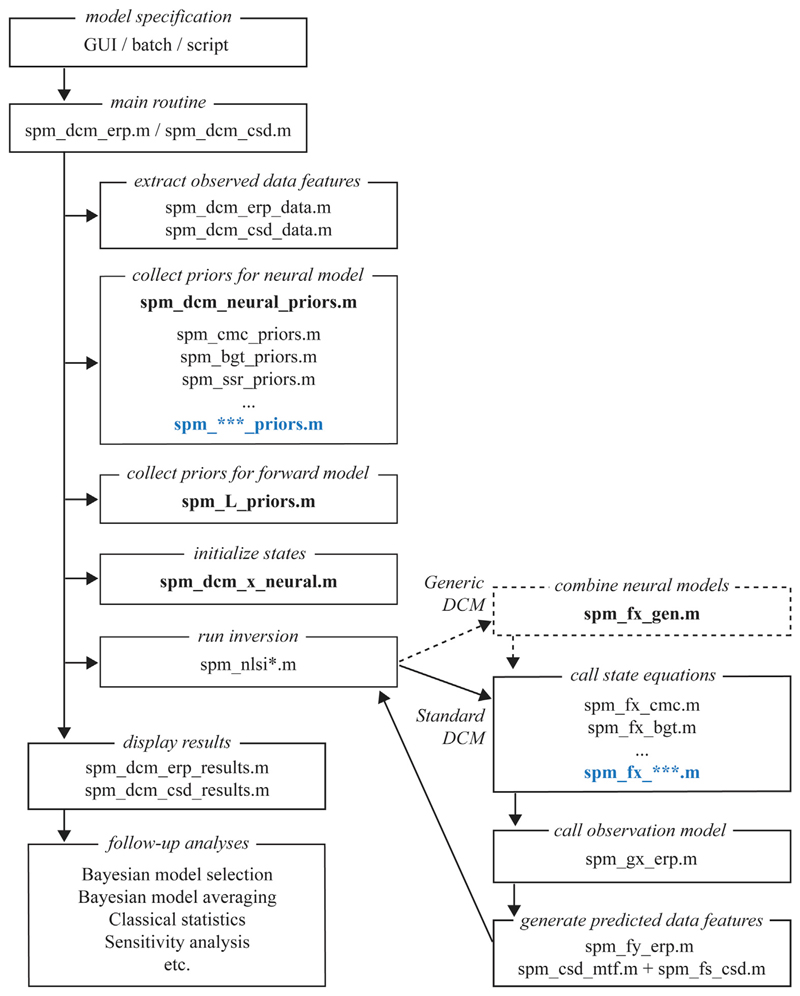
Simplified flow chart of the standard and generic DCM implementations. The main difference between the implementations is the addition of spm_fx_gen.m for generic DCM, which gathers the intrinsic (within-source) state dynamics for the different types of neural mass models in the network and adds extrinsic (between-source) coupling. Currently the generic implementation can only be called using script-based model specification. Addition of new neural mass models to the existing suite of models and their integration within the existing Bayesian inversion scheme is relatively straightforward. New functions that should be created for additional neural mass models are highlighted in blue and those that should be modified are indicated in bold.

DCM is implemented in the Matlab-based open-source SPM (‘Statistical Parametric Mapping’) software that can be downloaded from http://www.fil.ion.ucl.ac.uk/spm/. It can be operated via a graphical user interface, in batch mode, or by calling the relevant Matlab functions directly in a script. In this section, we describe the standard implementation before detailing our changes in the next section. The DCM pipeline is largely independent of neuroimaging modality, data feature, and choice of neural mass model. The specification of the generative model is fully separate from the inversion scheme and follows a standard format. The core of each neural model is formed by an spm_fx_***.m function describing the equations of motion. These have parameters that are specified in terms of prior means and variances in spm_***_priors.m. These two functions are hence unique for each type of neural mass model. In addition, an observer function maps neuronal states at the source level to recorded signals at the sensor level. This entails a scaling of depolarisation in (a mixture of) neural populations and multiplication with a conventional forward (leadfield) model (**spm_gx_erp.m**). Subsequently, data features in the form of event-related potentials (ERP) or cross-spectral densities (CSD) are generated via **spm_fy_erp.m** and **spm_fs_csd.m**, respectively, where spectral responses are obtained via the system's transfer functions in **spm_csd_mtf.m**. Prior distributions for the parameters used in these observation functions are specified in **spm_L_priors.m** and **spm_ssr_priors.m**. In order invert a DCM, users first specify the model options – and network structure – in the graphical user interface (as a batch, or in a custom script) before calling one of the inversion routines **spm_dcm_erp.m** or **spm_dcm_csd.m**, depending on the data feature of interest. This automatically collects the appropriate data features and prior distributions, sets the initial states, and calls the inversion scheme spm_nlsi*.m. After inversion, additional functions can be used, e.g., to visualize results and perform model comparisons. The entire pipeline is presented in Fig. 2.

### 2.2 Generic DCM implementation

In order to couple sources that differ in their intrinsic (within-source) dynamics, a new function **spm_fx_gen.m** has been introduced that serves as a parent routine that calls the state equations for each individual model type within the network, and adds the contribution of extrinsic (between-source) connections. The only change, from the perspective of the user, is the specification of model type for each source separately, which is now encoded in separate structures. Table 1 illustrates the exact format. A field is included to specify which intrinsic connections are free to vary between conditions (fixed in the standard implementation). Another new option is the direct specification of the hidden state(s) that contribute to the measured signal. This is useful for models like the basal ganglia-thalamus model, where it is possible for studies to use recordings from different anatomical structures. As before, after specification of the DCM, a call is made to either **spm_dcm_csd.m** for spectral data features or **spm_dcm_erp.m** for time domain data features. An example script is available under the *example_scripts* folder within SPM or upon request. We have also included the documentation for the generic prescription in Appendix 1.

In principle, the current implementation of the generic DCM scheme could support the composition of any neural mass or neural field sources to create a model of distributed neuronal responses. Having said this, the practical implementation requires one to distinguish between extrinsic (between-source) and intrinsic (within-source) coupling. The extrinsic coupling clearly has to be conserved in its form over sources. At present, a parameterised sigmoid activation (i.e., voltage to firing rate) function is applied to specified hidden states of each source and the resulting spike-rates drive specified (usually conductance) hidden states in each source. The specification of efferent and afferent extrinsic effects is in terms of the indices of source specific hidden states. In short, the integration scheme assembles the intrinsic and extrinsic flows separately, where the extrinsic flows have the same form. This formal constraint should, in principle, accommodate both convolution and conductance based intrinsic models; however, at present only convolution models are accommodated.

Generic DCM facilitates source-specific model specification via DCM.options.model(*n*), which should be specified for each source (*n* = *1* … *N*) in the network. This includes an option to specify which intrinsic connection strengths vary between conditions (field B), and an option to indicate which neural states contribute to the observed signal (fields J and K) in cases that differ from the default priors. Abbreviations of data features: ERP (Event-Related Potential), CSD (Cross-Spectral Density). Abbreviations of neural mass models: ERP (Event-Related Potential), CMC (Canonical Microcircuit Model), MMC (Motor cortex Microcircuit Model), BGT (Basal Ganglia-Thalamus Model), NFM (Neural Field Model), NMM (Neural Mass Model). For a complete list of currently available models see Table 2. Abbreviations of spatial models: ECD (Equivalent Current Dipole), IMG (Imaging), LFP (Local Field Potential). Additional (less commonly used) options are listed in the user documentation of the DCM Matlab functions.

### 2.3 Addition of new models

The procedure for adding new neural mass models and integrating them with existing ones is relatively straightforward. This enables users to contribute models for brain regions that are not adequately described by current models; for example, the cerebellum, hippocampus, or even the spinal cord. Minimal additions of new functions and changes to existing ones are required. The first step is the creation of an spm_fx_***.m function containing the state equations of the new source model, typically based on previous anatomical and physiological experimental work. This should be accompanied by an spm_***_priors.m function containing the prior distributions of model-specific neural parameters. Information about the new neural mass model should subsequently be added to **spm_dcm_neural_priors.m** (for selecting the appropriate prior function), **spm_L_priors.m** (for describing the lead field mapping between the model's hidden states and the measured signals), and **spm_dcm_x_neural.m** (for setting the number of states and their initial values). Finally, the input and output cell populations for extrinsic connections, as well as the expected intrinsic delays should be specified in **spm_fx_gen.m**. Fig. 2 illustrates the role of these functions in the DCM pipeline.

### 2.4 A cortico-basal ganglia circuit

We illustrate the use of generic DCM by coupling a motor cortex microcircuit model and a basal ganglia-thalamus model comprising four main basal ganglia structures and the thalamus. The architecture of the combined model is described in this section – and its application to study the effect of dopaminergic medication on beta oscillations in Parkinson's disease is presented in the next section. Both the motor cortex microcircuit (Bhatt et al., 2016) and the basal ganglia model (Moran et al., 2011a; Marreiros et al., 2013) have been used in previous publications using custom-written code. Here, we make these models publicly available by integrating them within the generic DCM framework.

The motor cortex microcircuit model (MMC) is based on adaptations to the canonical microcircuit model and subsequent Bayesian model comparison (Bhatt et al., 2016). These modifications have been applied to account for cytoarchitectonic differences between the primary motor cortex and especially visual cortex (Shipp, 2005; Beul and Hilgetag, 2015), upon which the canonical microcircuit model is based. Although primary motor cortex is known for being agranular, recent work nevertheless provides evidence that pyramidal cells located at the border between layer 3 and 5a possess classical layer 4-like properties (Yamawaki et al., 2014). The model therefore includes a separate middle-layer pyramidal cell population in addition to the superficial and deep populations. A single interneuron population accounts for unspecific inhibitory input across all layers (Fino et al., 2013). Excitatory interlaminar connections are primarily based on *in-vitro* photo-stimulation studies in mice (Weiler et al., 2008; Anderson et al., 2010; Hooks et al., 2011). Connections for which biological evidence was ambiguous were included or eliminated based on model comparisons (Bhatt et al., 2016).

The basal ganglia - thalamus model (BGT) was constructed to study the emergence of beta oscillations in 6-OHDA-lesioned rats (Moran et al., 2011a) and human Parkinson's disease patients with implanted deep brain stimulation electrodes (Marreiros et al., 2013). The model comprises five subcortical structures: striatum (Str), external segment of the globus pallidus (GPe), subthalamic nucleus (STN), internal segment of the globus pallidus (GPi) and motor thalamus (Tha). Interconnectivity between structures is based on the known main GABAergic and glutamatergic projections (Smith et al., 1998; Bolam et al., 2000) and encompasses the direct pathway (Str – GPi – Tha) as well as the indirect pathway (Str – GPe – STN – GPi – Tha). In addition, the model incorporates the glutamatergic feedback connection from STN to GPe, which might have a critical role in generating beta oscillations (Bevan et al., 2002). Each structure is represented by a single population of either excitatory or inhibitory neurons. Different types of interneurons make up ~5% of the striatum (Gerfen and Wilson, 1996) and were grouped into a single inhibitory self-connection. Pallidal inhibitory self-connections were added to reflect local axon collaterals (Kita and Kita, 1994; Sato et al., 2000; Sadek et al., 2007).

The MMC and BGT nodes are coupled via extrinsic excitatory connections. We included the projection from deep pyramidal cells to striatum (Cowan and Wilson, 1994) as well as the hyperdirect pathway connection to the subthalamic nucleus (Nambu et al., 2002). Thalamocortical projections originating from motor thalamus (ventrolateral nucleus) have been found to project to pyramidal cells in both layer 5b and layer 4 (Yamawaki et al., 2014) and were both modeled. In keeping with the other DCM models and based on the evidence for a presumed layer 4 (Yamawaki et al., 2014), we modeled these as endogenous input to layer 4. Connections from pre-motor and pre-frontal areas primarily target deep pyramidal cells with a less strong innervation to superficial layers (Hooks et al., 2013). In addition, we included a constant drive to primary motor cortex representing general thalamic and sensory input, which targets most strongly the layer 3/5a border (Mao et al., 2011; Hooks et al., 2013; Hunnicutt et al., 2014). A constant drive to striatum was also included to reflect input from premotor and somatosensory areas not included in the network.

### 2.5 Neuronal dynamics

The neuronal state equations describe the dynamics of a population's membrane potential in response to synaptic input through the convolution-based operation *v_post_* = *h* ⊗ *S*(*v_pre_*), Where *S* is a sigmoidal function translating pre-synaptic membrane potential into firing rate, and $h(t)=\frac{t}{T}e^{-\frac{t}{T}}$ for *t* ≥ 0 and *h*(*t*) = 0 for *t* < 0 is a synaptic kernel converting pre-synaptic firing rate into post-synaptic membrane potential (Jansen and Rit, 1995; David et al., 2006, Moran et al., 2007; 2013). The magnitude of this response is scaled by the synaptic coupling strength. This can be written as the following second order differential equation:

$${\ddot{v}}_{j}^{k}(t)=\left(\gamma_{l}^{k}S(v_{l}^{k}(t))+A_{l}^{m}S(v_{l}^{m}(t))+I^{k}(t)-2{\dot{v}}_{j}^{k}(t)-\frac{v_{j}^{k}(t)}{T_{j}^{k}}\right)/T_{j}^{k}$$

**Fig. 3:**
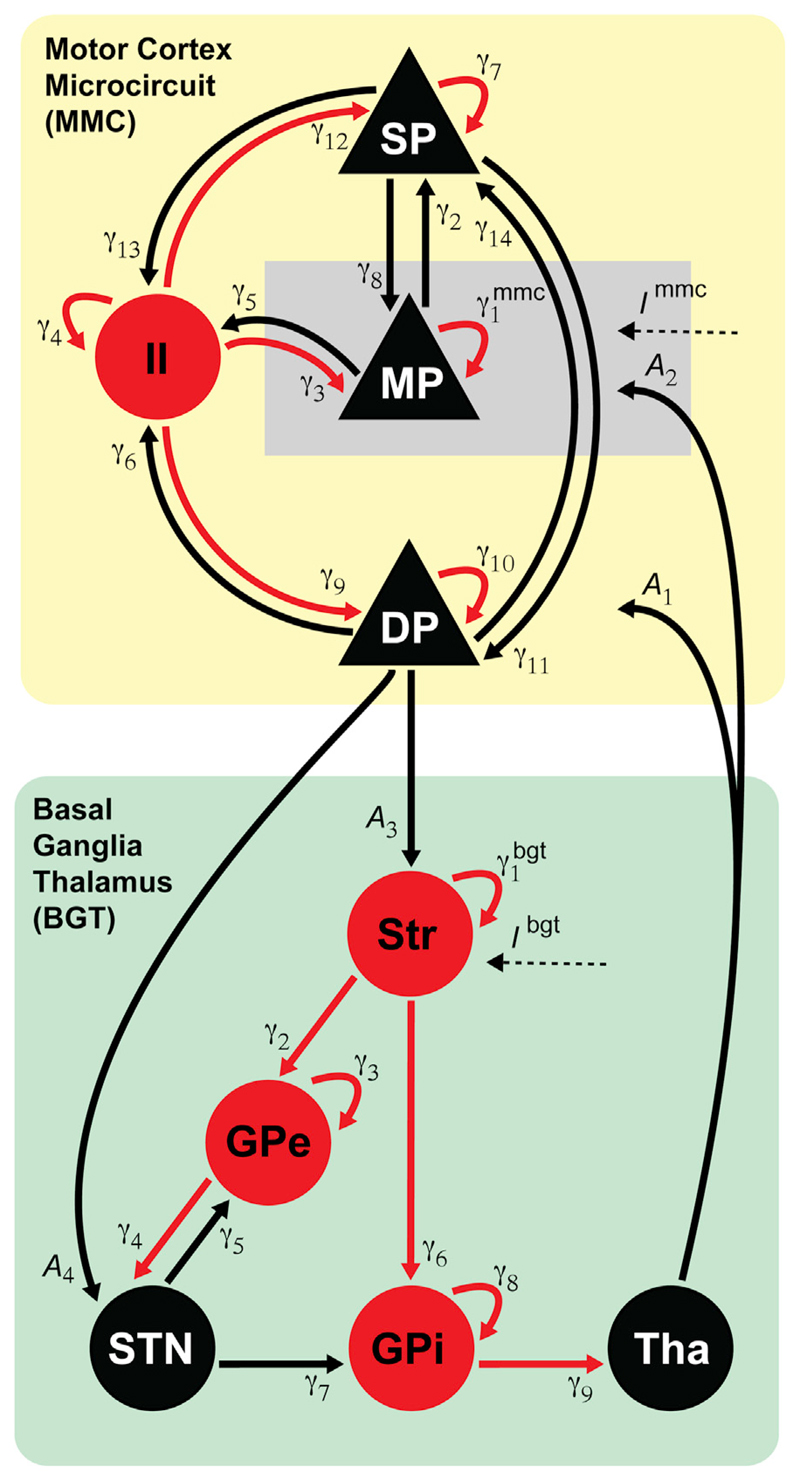
Network architecture of the cortico-basal ganglia circuit. Motor cortex (MMC model) and basal ganglia - thalamus (BGT model) are implemented as two separate sources coupled via extrinsic connections (*A*_1…4_). Intrinsic connections reflect synaptic coupling strengths between cell populations within motor cortex $\gamma_{1\ldots 14}^{mmc}$ and between basal ganglia structures and thalamus $\gamma_{1\ldots 9}^{bgt}$ Endogenous input in the form of colored noise enters the pyramidal cells in the middle layer of the motor cortex and the basal ganglia at the level of the striatum. Excitatory cell populations and connections are shown in black, inhibitory populations and connections in red. SP = superficial layer pyramidal cells; MP = middle layer pyramidal cells; DP = deep layer pyramidal cells; II = inhibitory interneurons; Str = Striatum; GPe = globus pallidus external segment; STN = subthalamic nucleus; GPi = globus pallidus internal segment; Tha = thalamus.

Membrane potential *v* of cell population *j* in source *k* is influenced by cell populations *l* within the same source with coupling strength $\gamma_{l}^{k}$ and with coupling strength $A_{l}^{m}$ from other sources *m*. Excitatory connections have positive coupling strength values and inhibitory connections negative. The membrane time constant $T_{j}^{k}$ is unique for each population. The sigmoidal function is denoted as $S(v)=\frac{1}{1+e^{-Rv\cdot }}$. Its slope is parameterised by *R* and captures the variability in response properties within a cell population. The deviation in firing rate from baseline firing (obtained for *v* = 0) is converted into post-synaptic membrane potential. Finally, endogenous input *I^k^* is modeled as colored noise to reflect the scale free (1/f-like) spectrum of endogenous neural activity (generated by brain regions outside the specified network). Scale free fluctuations mean that the relationship between the amplitude of fluctuations and their frequency can be expressed as a power law, characterised by a scaling exponent: *ψ_u_* = *α*_*u*_*ω*^−*β*_*u*_^, where we use subscript *u* to distinguish this input from observation noise of the same form (see below). Fig. 3 depicts the model's connectivity structure and the populations receiving endogenous input. Compared to (Moran et al., 2011a; Marreiros et al., 2013), we absorb maximum excitatory/inhibitory rate constants into our synaptic connection strengths *γ*, to ensure the BGT is formally consistent with the MMC. Time delays within and between sources are not explicitly incorporated in the state equations but instead implemented via a Taylor series approximation of the Jacobian matrix (see Appendix A.1 of David et al., 2006).

The neuronal state equations are supplemented by an observation function, mapping hidden neural states to the measured signals. For the MMC source, we fixed observed signal to be a mixed contribution of [0.2 0.2 0.6] from superficial, middle, and deep pyramidal cells. For the BGT source, the observed signal was set to come from the STN. The scaled contribution of each source to the measured signal is encoded by the lead field matrix *L*. In case of LFP recordings or source-extracted data this is a mere gain function. At this point in the forward modelling, observation noise common (subscript *c*) to recordings from motor cortex and the STN and channel-specific noise (subscript *s*) are also added to the spectral responses predicted by the model; again in the form of colored noise *ψ_c_* = *α_c_ω*^−*β_c_*^ and *ψ_s_* = *α_s_ω*^−*β_s_*^ (Moran et al., 2009).

All free parameters and their prior distributions are summarized in Table 3. Nonnegative parameters (such as time constants) are implemented as exponential scale-factors of their prior means. The priors in Table 3 therefore have a lognormal distribution with an expectation of zero. As we are working with a new type of DCM model, we ensured that model inversion relied more heavily on achieving accurate fits than on prior expectation values by increasing the expected precision *hE* of the observed data and choosing relatively broad prior variances.

## 3 An empirical example

We used the cortico-basal ganglia circuit model of the previous section to infer alterations in synaptic coupling strength underlying the reduction in STN beta oscillations observed in Parkinson's disease patients following dopaminergic medication.

### 3.1 Experimental data

The data set we used here forms a subset of data used in previous studies (Litvak et al., 2011; van Wijk et al., 2016). The patients who participated were diagnosed with Parkinson's disease according to the Queen Square Brain Bank Criteria (Gibb and Lees, 1988) and underwent surgical implantation of deep brain stimulation electrodes in left and right subthalamic nucleus at the National Hospital of Neurology and Neurosurgery (University College London) following the center's standard procedures (Foltynie et al., 2011). Each electrode lead (model 3389, Medtronic, Minneapolis, MN, USA) contained four macro-electrode contacts of 1.5 mm diameter that were spaced 2 mm apart (center-to--center). The center of the STN was determined as the surgical target for the lowermost contact as identified on a pre-operative stereotactic axial T2-weighted MRI scan at the level of the largest diameter of the red nucleus and 0–1 mm behind its anterior border (Bejjani et al., 2000). 11 Patients (2 female) were included in this study. Their mean age (±sd) at the time of recordings was 54.6 ± 6.1 (range 40–61) years, with a disease duration of 12.2 ± 2.9 (range 8–17) years. United Parkinson's Disease Rating Scale (UPDRS) hemibody subscores for bradykinesia and rigidity were off medication 11.5 ± 5.4 (range 5–23), and 3.2 ± 1.8 (range 0–6) on medication.

Within 2–7 days after implantation, simultaneous magnetoencephalography (MEG) and local field potential (LFP) recordings from STN were obtained in two separate sessions on subsequent days. In random order, one of the sessions was performed whilst the patient was ‘ON’ their regular dose of dopaminergic medication, the other session after overnight withdrawal (‘OFF’). Signals were low-pass filtered at 600 Hz and sampled at 2400 Hz. An offline bipolar derivation was applied between adjacent LFP contact pairs, resulting in three time series per STN. All patients with both ON and OFF recordings available were considered in the present study. Ethical approval was obtained from the local ethics committee and all patients gave written informed consent prior to the recordings.

Our analyses are based on resting state recordings of about 3-min duration. The continuous data were cut into 3.41s epochs. Trials with STN-LFP or MEG source-extracted amplitude values exceeding 7 standard deviations of the entire time series were discarded, leaving on average 46 ± 15 trials (range 16–88) per condition for each hemisphere. Data from one hemisphere had to be excluded because of poor STN recordings in the OFF condition in which none of the trials survived the artifact rejection criteria. In our previous work, we used DICS beamforming to identify the motor cortical source with largest resting state beta band coherence (15–35 Hz) with each STN-LFP time series (Litvak et al., 2011). We selected per hemisphere the LFP contact pair with largest beta band coherence and used the beamformer weights for the corresponding source location to construct a ‘virtual electrode’ comprising the motor cortical source time series. This was necessary to suppress artefacts in the MEG originating from the percutaneous wires that were attached to the deep brain stimulation electrodes (Litvak et al., 2010). Hence, for each hemisphere, we have one STN time series and one motor cortical time series (divided into epochs). Auto- and complex cross-spectral densities were computed using Bayesian multivariate autoregressive modelling (Roberts and Penny, 2002) with order 12 for frequencies between 5 and 45 Hz. These served as the data features to be predicted by the DCM model (Friston et al., 2012).

### 3.2 Model inversion

The objective of model inversion is to find posterior parameter densities that provide the most accurate explanation of observed data features, while minimizing the model's complexity (i.e., deviation from prior distributions). In DCM for complex cross spectral densities, model predictions are generated via a kernel response to endogenous input (innovations) in the spectral domain (Moran et al., 2007; Friston et al., 2012). The model's connectivity structure, lead field matrix, and parameter values constitute the system's transfer functions (one per endogenous input source and data channel), which are multiplied with the spectral density of the innovations to generate predicted auto- and complex cross-spectra that are to be compared with the observed spectra. Parameter expectations and precisions are updated via Variational Bayesian inference under the Laplace approximation of Gaussian posterior density distributions. This Variational Laplace scheme generalizes the coordinate ascent expectation-maximization algorithm (Friston et al., 2007). The objective function is variational free energy, which serves as an approximation (i.e., lower bound) to the log-model evidence (Friston et al., 2006, 2007; Friston, 2010).

Given the novel character of our cortico-basal ganglia circuit, we first determined appropriate prior means for synaptic coupling strengths (intrinsic and extrinsic) and population time constants by fitting the model to grand-averaged spectral densities. We explored a range of initial values that were variations on prior values previously used for the BGT and MMC and the CMC model.^1^ In DCM, multiple conditions can be modeled simultaneously by including a set of *B* parameters that represent the difference in synaptic coupling strengths from a baseline or control condition. We always modeled the OFF medication state as a baseline condition and ON medication as trial-specific effects on all synaptic coupling strengths (*B*). Posterior means for the ON condition are hence obtained by adding the *B* estimates to *γ* or *A*; i.e., baseline intrinsic or extrinsic connectivity. As the inversions were prone to early convergence, we re-initialized each of them several times (re-initializing with posterior estimates) to preclude local minima solutions.^2^ Posterior means for synaptic coupling and time constants obtained for the inversion with most accurate auto- and cross-spectral densities were taken as prior values for the individual inversions described below. Data from one subject with exceedingly strong beta oscillations (spectral peak amplitude larger than 5 standard deviations above the group mean) were left out of the grand-average, in order to obtain more representative group-level spectral densities; however, this subject was included in the individual inversions.

**Fig. 4:**
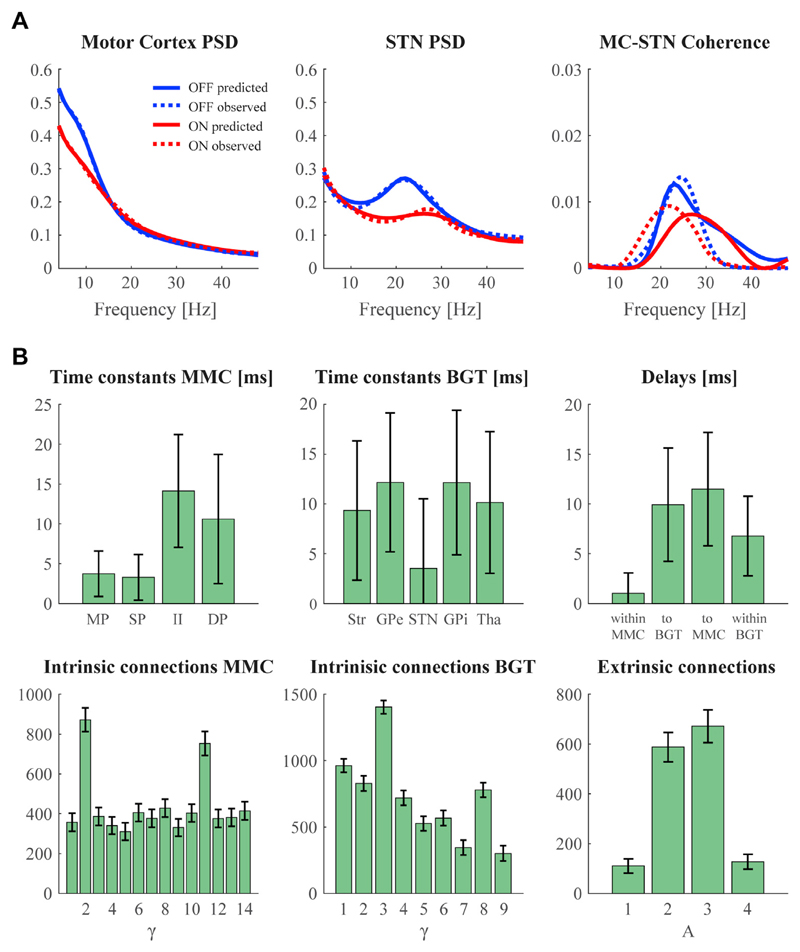
Model inversion results for the grand average data. Panel A shows the model's predicted power spectral densities (PSD) and coherence overlaid on the observed spectra. Panel B shows the corresponding posterior means of the baseline (OFF medication) condition. See Fig. 3 for the correspondence between index numbers and anatomy, and abbreviations of cell populations. The bars denote the 95% Bayesian confidence (or credible) intervals based upon posterior covariance estimates.

**Fig. 5:**
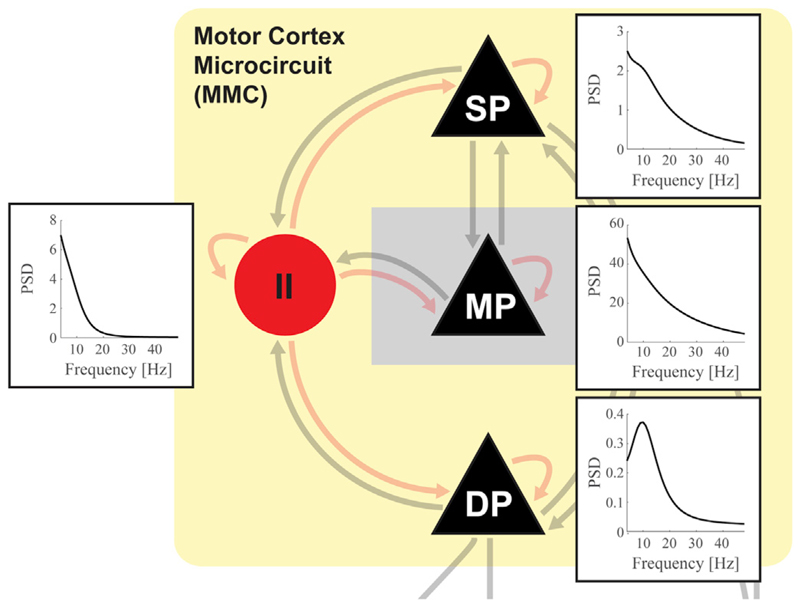
Maximum a posteriori (MAP) estimates of [auto] spectral responses in layer-specific neural populations. Results are based upon the MAP estimates of the underlying synaptic and connectivity parameters in the OFF medication condition. Effectively, these are obtained by running DCM in a forward modelling mode, using a lead field that plays the role of a virtual electrode; sampling each population (in the absence of channel noise).

Model predictions for the inversion that most accurately captured the grand-average spectra are presented in Fig. 4A, where we display the complex-valued cross-spectrum as coherence. There is a close match between predicted and observed spectral densities for the motor cortex and the STN, including the suppression of a clear beta peak in STN after dopaminergic medication. Cross-spectral density values between motor cortex and STN were much lower compared to the auto-spectra but were still adequately predicted by the model with a distinct peak in the beta frequency range for both conditions. The most relevant parameter estimates resulting from this inversion are presented in Fig. 4b. There are a few things of interest to note here. First of all, the time constants of the neural populations in the MMC model could support a dissociation between fast activation in superficial layers versus slower activation in deep layers, as observed by layer-specific oscillation frequencies in experimental recordings (Roopun et al., 2006; Buffalo et al., 2011). To quantify laminar-specific spectral responses in our network, we computed the auto-spectrum of each cortical population from the system's Jacobian at the *maximum a posteriori* (MAP) estimates. By specifying a lead field that samples each population, the associated MAP estimates of spectral responses can be evaluated in the usual way. This revealed that deep layers displayed relatively strong low-frequency (alpha) activity – see Fig. 5. Note that high-frequency activity is not produced by the individual cortical populations as it is not apparent in the observed data.

Secondly, STN neurons are known to respond relatively fast to input (Farries et al., 2010), which is reflected by a lower time constant compared to the other basal ganglia nuclei. Pallidal and striatal time constants are close to experimentally observed membrane time constants as summarized in a meta-analysis (http://neuroelectro.org). Furthermore, stronger synaptic coupling strengths were assigned to the dominating cortical pathways from layer 4 to 2/3 and layer 2/3 to 5 (Weiler et al., 2008; Yamawaki et al., 2014). Likewise, the corticostriatal projection was stronger than the hyperdirect pathway. We leave the discussion of medication-induced changes to the next section, where we describe results based on individual inversions. Full details of prior distributions for these are listed in Table 3.

Prior means for intrinsic and extrinsic coupling strengths (*γ*, *A*), as well as time constants (*T*) were taken from the posterior means obtained after model inversion of the grand average spectra. Other prior means remained at their original values. Parameters are generally implemented as exponential scaling factors of the prior expectations to ensure non-negativity constraints: *ϑ_i_* = *π_i_e^θ_i_^*, with $\theta_{i}=𝒩(0,\sigma_{i}^{2}),\pi_{i}$ is the prior expectation and $\sigma_{i}^{2}$ its log-normal dispersion. Wider distributions were used for the BGT model to accommodate our uncertainty about their values. See Fig. 3 for the correspondence between index numbers and anatomy, and abbreviations of neural populations.

### 3.3 Group-level parameter inference

The origin of oscillations within the basal ganglia has been the focus of various experimental studies. On the one hand, much emphasis has been placed on the recurrent excitation-inhibition circuitry between STN and GPe, which has the natural capacity to produce oscillations (Bevan et al., 2002). Indeed, it has been shown that the STN-GPe circuit *in vitro* shows synchronized low-frequency oscillatory bursting behaviour (Plenz and Kital, 1999). Furthermore, lesions or blocked synaptic input within the STN-GPe circuit disrupt the oscillations (Ni et al., 2000; Tachibana et al., 2011). On the other hand, other evidence points towards a cortical origin. Directionality analysis between simultaneously recorded MEG and STN-LFPs indicates a leading role for cortex in the beta range (Williams et al., 2002; Fogelson et al., 2006; Litvak et al., 2011; Oswal et al., 2016). Cortical beta oscillations could reach the STN via the hyperdirect or the indirect pathway. In the latter case, D2-expressing medium spiny projection neurons (D2-MSN) may become more sensitive to cortical input in the dopamine depleted state, leading to an over-activation of the indirect pathway and hence a larger influence of cortical activity on the basal ganglia (Brown, 2007; Kreitzer and Malenka, 2009; Weinberger and Dostrovsky, 2011). These mechanisms of beta generation are not mutually exclusive.

**Fig. 6:**
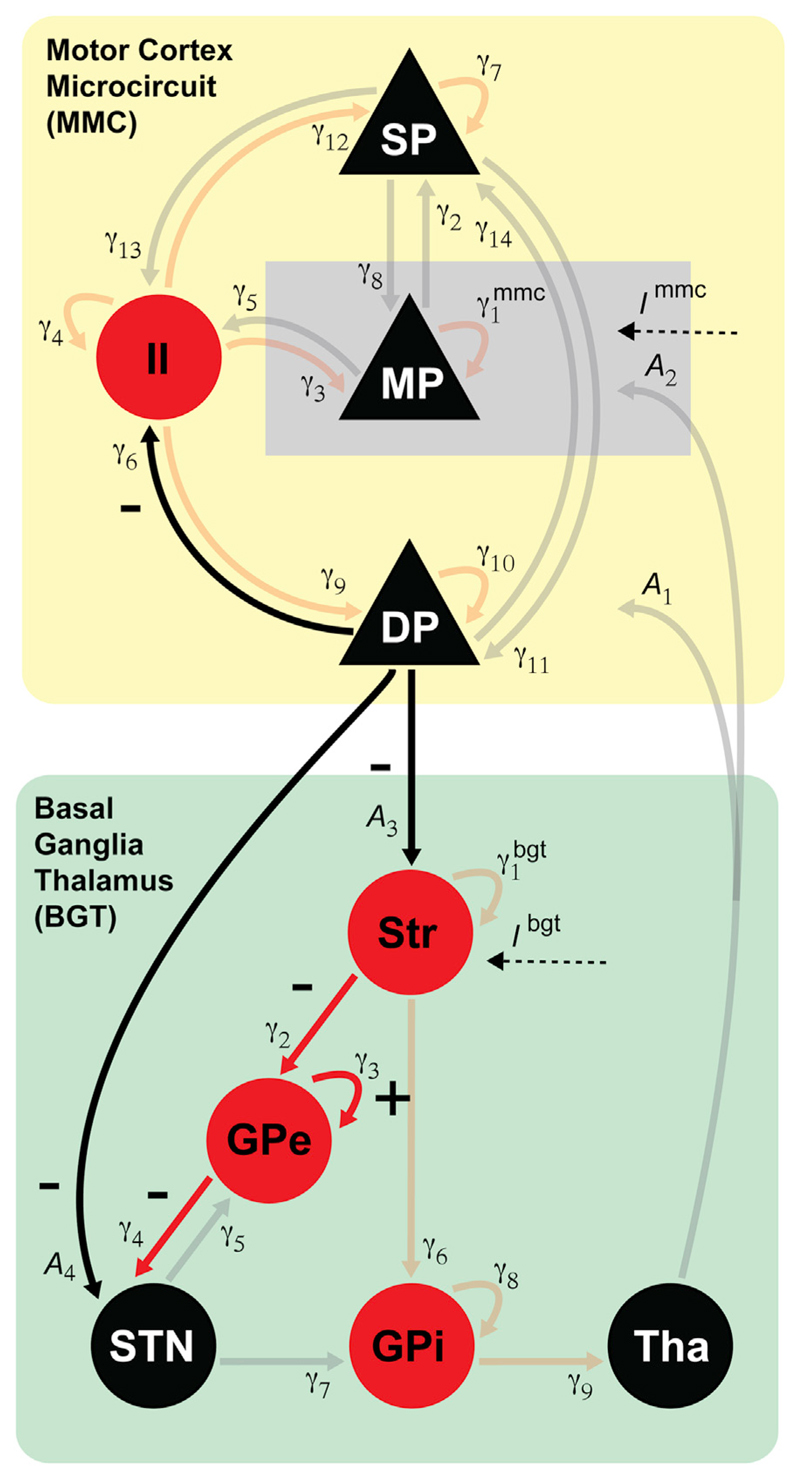
Group level inference on medication-induced changes in synaptic efficacy. Connections with significantly altered coupling strength between ON and OFF medication conditions are indicated in bold. Corresponding ‘+’ and ‘-’-signs indicate whether medication increased or decreased the posterior mean of the connections.

To identify which synaptic connections in our network were altered by dopaminergic medication, we inverted the model for each hemisphere individually. For one hemisphere in one subject we were unable to obtain an adequate model prediction of the observed spectra (spectral predictions remained flat). This hemisphere was omitted from further analyses; hence, the individual inversions resulted in 20 sets of posterior mean values. As we were interested in alterations of synaptic strength between conditions, we only further considered the (*B*) parameters encoding changes in intrinsic and extrinsic connectivity. For each connection we performed a *t*-test against zero to test for a significant difference between conditions over subjects. This revealed a significant decrease in synaptic coupling strength (efficacy) following dopaminergic medication for the corticostriatal projection (*t*(19) = −2.42, *p* = .026), the hyperdirect pathway (*t*(19) = −3.14, *p* = .005), the connection from striatum to the external pallidum (*t*(19) = −2.57, *p* = .019), from the external pallidum to STN (*t*(19) = −2.54, *p* = .020), and the cortical connection from infragranular pyramidal cells to inhibitory interneurons (*t*(19) = −2.96, *p* = .008). A medication-induced increase in connection strength was only found for inhibitory self connections of the external pallidum (*t*(19) = 2.58, *p* = .018). Nevertheless, none of these *p*-values survived significance after a false discovery rate correction for multiple comparisons. Results are shown in Fig. 6.

## 4 Discussion

Many human electrophysiological studies simply describe how certain EEG/MEG data features change with behavioral tasks, cognitive states, and pharmacological interventions or differ between patient groups and healthy controls. In contrast, analyses based on forward or generative models – such as DCM – try to identify the neural origin of these effects by linking experimental recordings to synaptic activities. In this paper, we have presented a generalization of DCM that affords greater latitude in its applications. Most importantly, it allows for the combination of network nodes or sources that differ in intrinsic architecture. We illustrated this flexibility by coupling a motor cortex microcircuit model with a basal-ganglia-thalamus model, and used the resulting DCM to ask how dopaminergic medication leads to a reduction in beta oscillations in Parkinson's disease. We found evidence for weaker synaptic efficacy within the STN-GPe circuit, as well as weaker hyperdirect and indirect pathway connections.

The implementation of DCM in SPM has gradually been improved and extended over recent years. At the time of writing, it contains a fairly broad suite of neural mass and field models that have been designed to reflect the canonical architecture of the cortex (see Table 2). However, the standard implementation only permits one type of model in each inversion. The same model, therefore, has to be used for each node (or ‘source’) in the network. While this serves the majority of EEG/MEG studies with merely cortical nodes, it is less suited for networks involving sources that do not adhere to a laminar organization, like the many subcortical regions, cerebellum, or spinal cord. Even regional variations in cortical anatomy can be a motivation for adjustments to the canonical models, as exemplified by the motor cortex microcircuit model. The generic DCM framework facilitates these non-standard applications by allowing for a more flexible composition of distributed sources.

In virtue of specifying the model dynamics in terms of equation of motion, the current scheme restricts generic DCM to state space models that can be specified as ordinary differential equations (ODEs). This precludes the direct use of models specified as delay differential equations (DDE), partial differential equations (PDE), integro-differential equations or stochastic differential equations (SDE) with additive or multiplicative noise. However, in many cases one can reduce more elaborate models to an ODE. In DCM for EEG, high-order Taylor approximations are used to convert DDEs into ODEs. Indeed, the delays are a free parameter of the DCM: See the appendix of (Bastos et al., 2015) for a recent technical discussion. Similarly, it is possible to convert integro-differential equations associated with neural field models into ordinary differential equations using spatial modes: see for example (Pinotsis et al., 2014). Stochastic differential equations can be formulated in terms of their density dynamics using (Laplacian) approximations and the Fokker Planck formalism; see for example (Marreiros et al., 2009; Moran et al., 2013). Finally, stochastic dynamics can be converted into deterministic dynamics by using generative models of second order statistics; such as DCM for cross spectral density of the sort we have used here (Friston et al., 2012). Effectively, this converts stochastic fluctuations in time into the second order statistics of cross covariance functions or, in the frequency domain, the spectral behaviour of noise; e.g., the scale-free fluctuations used above.

In terms of practical constraints on the number of nodes (i.e. sources and constituent neural masses) in a DCM, there are a number of considerations. First, the computational cost of estimating large models increases with the number of sources. This reflects the fact that the free energy gradients, with respect to the number of free parameters, grows quickly with the number of sources. Having said this, the number of parameters can be surprisingly large; sometimes several hundred. Furthermore, increasing the dimensionality of parameter space can, perhaps counterintuitively, nuance the problem of local extrema. One perspective on this phenomenon is that adding extra parameters destroys local extrema (for example, adding an extra dimension to a minimum can convert it into a saddle point). Usually, DCM is used to answer specific questions (e.g., about condition or diagnosis effects) using carefully designed experiments that call for a small number of sources (e.g., between two and eight). As a rule of thumb, a typical DCM can normally be inverted on a personal computer within a few minutes, with convergence after about 16–64 iterations.

We have used an exemplar empirical application to demonstrate the ability of the generic framework to reproduce and substantiate findings from previous literature. The occurrence of strong beta oscillations in basal ganglia nuclei is a hallmark of Parkinson's disease (Gatev et al., 2006; Hammond et al., 2007; Oswal et al., 2013) and is indicative of the severity of motor impairments (Neumann et al., 2016; van Wijk et al., 2016). Identifying the synaptic circuits involved in beta generation is therefore of great importance in understanding the pathophysiology of movement disorders and development of targeted treatments. Our findings suggest that dopaminergic medication has a widespread effect on subcortical effective connectivity. This is to be expected as – in addition to the striatum – dopaminergic projections from substantia nigra innervate the pallidum and subthalamic nucleus (Cossette et al., 1999).

Empirical studies have shown that dopamine reduces the impact of GABAergic striatal inputs to GPe (Cooper and Stanford, 2001) and of GABAergic inputs to STN (Cragg et al., 2004). This support the results we observed here, as well as previous modelling work showing that the STN-GPe circuit is capable of inducing oscillations (Gillies et al., 2002; Terman et al., 2002; Humphries et al., 2006; Holgado et al., 2010; Pavlides et al., 2012; Liu et al., 2016) but with a critical influence of connections directly leading to the STN-GPe circuit (Gillies et al., 2002; Terman et al., 2002; Holgado et al., 2010; Kumar et al., 2011). Also the two previous DCM studies using a cortico-basal ganglia circuit found evidence for a contribution of both of STN-GPe connections and the hyperdirect and indirect pathway to the amplitude of beta oscillations (Moran et al., 2011a; Marreiros et al., 2013). Alternatively, oscillations might arise elsewhere in the cortical or cortico-thalamic system and propagate through to the basal ganglia (van Albada et al., 2009; Hahn and McIntyre, 2010; Pavlides et al., 2015). This scenario seems unlikely in our case as spectral beta peaks were not always observed in our MEG recordings, suggesting that excessive beta oscillations are primarily a subcortical phenomenon. However, we acknowledge that the lack of spectral beta peaks might be due to the lower signal-to-noise-ratio inherent to MEG recordings. Encouragingly, the use of ECoG during deep brain stimulation surgery is gaining interest in the field, which could help resolve the ambiguous role of cortical oscillations in Parkinson's disease (de Hemptinne et al., 2013; Kondylis et al., 2016).

Previous DCM work – with more phenomenological generative models – has examined levodopa-induced alterations in effective connectivity in Parkinsonian patients using fMRI (Michely et al., 2015; Rowe et al., 2010) and EEG (Herz et al., 2014a, 2014b). A common finding in these studies is an increase in inter-regional coupling to supplementary motor area with medication, which predicts the severity of levodopa-induced dyskinesia with high accuracy (Herz et al., 2015). Although these models lack biological detail in their neural state descriptions, they are capable of identifying key extrinsic effective connectivity changes. This was demonstrated recently in an advanced experimental set-up combining simultaneous optogenetic stimulation and fMRI recordings in mice (Bernal-Casas et al., 2017). Upon stimulation of D1-MSN neurons, DCM identified increased connectivity strength along the direct pathway connections from striatum to GPi and substantia nigra. Vice versa, the indirect pathway connection from STN to substantia nigra was found to be increased during D2-MSN stimulation. Promising advances in the use of neural mass models in DCM for fMRI might allow for pinpointing the underlying synaptic signaling more precisely in future studies (Friston et al., 2017).

The basal ganglia form a distributed and intricately connected network that is difficult to fully capture with electrophysiological recordings. Computational modelling could therefore be highly valuable in studying the functional roles of the direct, indirect and hyperdirect pathways. While we demonstrated an application to movement disorders, it is conceivable to use the same network architecture to address cognitive or affective functions that are known to be reliant on cortico-basal ganglia-thalamus interactions, such as reward-based decision making (Balleine et al., 2007), working memory (McNab and Klingberg, 2008), obsessive compulsive disorder (Graybiel and Rauch, 2000), habit formation and addiction (Yin and Knowlton, 2006), and many more (Middleton and Strick, 2000; Kotz et al., 2009; Maia and Frank, 2011). In humans, the opportunity to collect electrophysiological data from subcortical structures is afforded by implanted deep brain stimulation electrodes that are used for treatment of an increasing number of movement and cognitive disorders (Krack et al., 2010). A more extensive coverage of basal ganglia activity however might be reached with animal models, which would provide tighter constraints on model parameters.

The generic DCM framework is primarily aimed at advanced DCM users who might appreciate more flexible control over modulatory effects on intrinsic coupling parameters and/or who wish to couple cortical or subcortical sources with distinct microcircuit architectures. We have also described the MATLAB functions that need to be modified or created when adding a new type of neural mass model to the DCM repertoire. This has the advantage that existing Variational Laplace schemes in SPM could be readily accessed for model inversion, including supplementary tools for Bayesian model comparisons (Stephan et al., 2009; Penny et al., 2010), and the recently introduced Parametric Empirical Bayes approach for group inversion and between-group effects inference (Friston et al., 2016). The generic implementation therefore augments the scope of research questions that could be addressed with DCM using physiologically and anatomically realistic models.

## Supplementary Material

Appendix 1

## Figures and Tables

**Table 1:** List of user-specified options for DCM inversion.

Field	Description	Examples
**Standard DCM**		
DCM.options.analysis	Data feature to be modeled	'ERP', 'CSD'
DCM.options.model	Type of neural mass model	'ERP', 'CMC', 'MMC', 'BGT', 'NFM', 'NMM'
DCM.options.spatial	Type of spatial (forward) model	'ECD', 'IMG', 'LFP'
DCM.options.trials	Indices of trials (conditions)	[1 2]
DCM.options.Nmodes	Number of spatial modes to invert	8
DCM.options.D	Time bin decimation (down-sampling)	1
DCM.options.Tdcm	[start end] Time window in ms	[0 1000]
DCM.options.onset	Stimulus onset in ms – used in DCM for ERP	60
DCM.options.dur	Stimulus dispersion (standard deviations) in ms – used in DCM for ERP	16
DCM.options.Fdcm	[start end] Frequency window in Hz – used in DCM for CSD	[4 48]
**Generic DCM**		
DCM.options.model(*n*).source	Type of neural mass model for the *n*-th source	'ERP', 'CMC', 'MMC', 'BGT'
DCM.options.model(*n*).B	Index number of intrinsic connections exhibiting condition-specific effects (optional)	[2 3 4 7], [1 4 7 10], [1:10]
DCM.options.model(*n*).J	Index number of neural states that contribute to the measured signal. Sets their prior expectation to 1 (optional)	3
DCM.options.model(*n*).K	Index number of neural states for which their contribution to the measured signal is estimated from the data. Sets their prior variance to 1/32 (optional)	[1 7]
Other options as listed for the standard DCM implementation		

**Table 2:** Currently available neural mass and field models in DCM.

Acronym	Full name	Type	Specifics	Reference
ERP	Event-Related Potential	Convolution/Neural Mass	Original model with 3 cell populations	David and Friston, 2003
SEP	Sensory-Evoked Potential	Convolution/Neural Mass	Faster version of the ERP model	David and Friston, 2003
LFP	Local Field Potential	Convolution/Neural Mass	ERP model with recurrent inhibitory connections for modelling gamma oscillations	Moran et al., 2007
CMC	Canonical Microcircuit	Convolution/Neural Mass	4-population model with separate supra-and infragranular pyramidal cell populations	Bastos et al., 2012; Auksztulewicz and Friston, 2015
MMC	Motor Microcircuit	Convolution/Neural Mass	4-population model based on motor cortex anatomy	Bhatt et al., 2016
BGT	Basal Ganglia and Thalamus	Convolution/Neural Mass	Subcortical model including 4 basal ganglia structures and thalamus	Marreiros et al., 2013; Moran et al., 2011a
NFM	Neural Field Model	Convolution/Neural Field	3-population model with spatiotemporal dynamics	Pinotsis et al., 2012
NMM	Neural Mass Model	Conductance/Neural Mass	Conductance-based version of the ERP model	Marreiros et al., 2009; 2010
MFM	Mean Field Model	Conductance/Mean Field	Conductance-based version of the ERP model with second order statistics	Marreiros et al., 2009; 2010
NMDA	Mean Field Model with NMDA receptor	Conductance/Mean Field	Conductance-based version of the ERP model with NMDA receptor and second order statistics	Moran et al., 2011b
CMM	Canonical Mean Field Model	Conductance/Mean Field	Conductance-based version of the CMC model with second order statistics	
CMM_NMDA	Canonical Mean Field Model with NMDA receptor	Conductance/Mean Field	Conductance-based version of the CMC model with NMDA receptor and second order statistics	

**Table 3:** Prior distributions for all parameters in individual inversions.

Parameter	Description	Prior values *π*, *σ*^2^
$\gamma_{1\ldots 14}^{mmc}$	Synaptic coupling strengths cortex	[357 872 387 340 311 405 377 429 331 403 753 376 382 414],1/4
$T_{1\ldots 4}^{mmc}$	Time constants [ms] cell populations cortex: [MP, SP, II, DP]	[3.7 3.2 14.1 10.6],1/8
$\gamma_{1\ldots 9}^{bgt}$	Synaptic coupling strengths basal ganglia	[962 828 1403 719 526 568 345 780 301],1/2
$T_{1\ldots 5}^{bgt}$	Time constants [ms] cell populations basal ganglia: [Str, GPe, STN, GPi, Tha]	[9.3 12.2 3.5 12.1 10.1],1/4
*A*_1…4_	Extrinsic connections strengths	[110 588 672 127],1/4
$B_{1\ldots 14}^{mmc}$	Condition-specific effects on intrinsic coupling strengths cortex	[0 0 0 0 0 0 0 0 0 0 0 0 0 0],1/4
$B_{1\ldots 9}^{bgt}$	Condition-specific effects on intrinsic coupling strengths basal ganglia	[0 0 0 0 0 0 0 0 0],1/2
*B*_1…3_	Condition-specific effects on extrinsic coupling strengths: [Tha to MMC, MMC to Str, MMC to STN]	[0 0 0],1/4
*R*	Slope sigmoidal function: [MMC, BGT]	2/3,[1/32 1/16]
*d*_1…4_	Delays [ms]: [within MMC; from MMC to BGT; from BGT to MMC; within BGT]	[1 8 8 4],1/32
*α*_*u*_, *β*_*u*_	Endogenous input (innovations). *I* = 512 *ψ*_*u*_	[1 1],1/4
*α*_*c*_, *β*_*c*_	Channel unspecific observation noise	[1 1],1/4
*α*_*s*_, *β*_*s*_	Channel specific observation noise	[1 1],1/4
*L*	Observation gain: MMC, BGT	[1 1],4
*hE*	Precision of observed data	16,4

## Footnotes

1 Although not our focus, it should be noted that the optimisation of priors in DCM for neurophysiological data is an important issue. In principle, this could proceed by treating the priors over unknown parameters as part of model specification and then performing model comparison to identify the best priors in a quantitative sense. In practice, one usually inverts the data at hand using successive line searches through parameter space to optimise model evidence (as scored by the free energy). This usually goes hand-in-hand with an accurate fit; accounting for about 90% of the variance. A heuristic diagnosis of ‘apt’ priors can be convergence rate: one would normally hope to see convergence within 64 iterations of the variational scheme used in DCM; however, minor improvements can often be obtained after 128 iterations, where a minor improvement is a trivial increase in free energy (often less than about 1/8 nats).

2 Local minima can be an issue for the sorts of models typically used in DCM for EEG and MEG (especially models of complex cross spectral data features). This is because these DCMs are usually nonlinear in the parameters. Furthermore, nonlinearities can present brittle ‘inversion’ problems due to phase transitions (e.g., when the eigenvalues of a DCM Jacobian cross zero). This sort of brittleness is finessed in DCM by detecting and precluding positive eigenvalues; however, the problem of local minima can still persist. One simple approach to this is to use a multi-start scheme; in other words, repeat the inversion from multiple initial estimates of the parameters: in our illustrative example we used a multi-start scheme by reinitialising the inversion with the MAP estimates of the parameters (but not the precision or hyperparameters) until convergence. This was repeated eight times.
