# Supplementary figures and images for: Generic dynamic causal modelling: An illustrative application to Parkinson's disease
Source: Neuroimage. Author manuscript; Available in PMC 2020 Jul 8. (PMC7343527; doi:10.1016/j.neuroimage.2018.08.039)

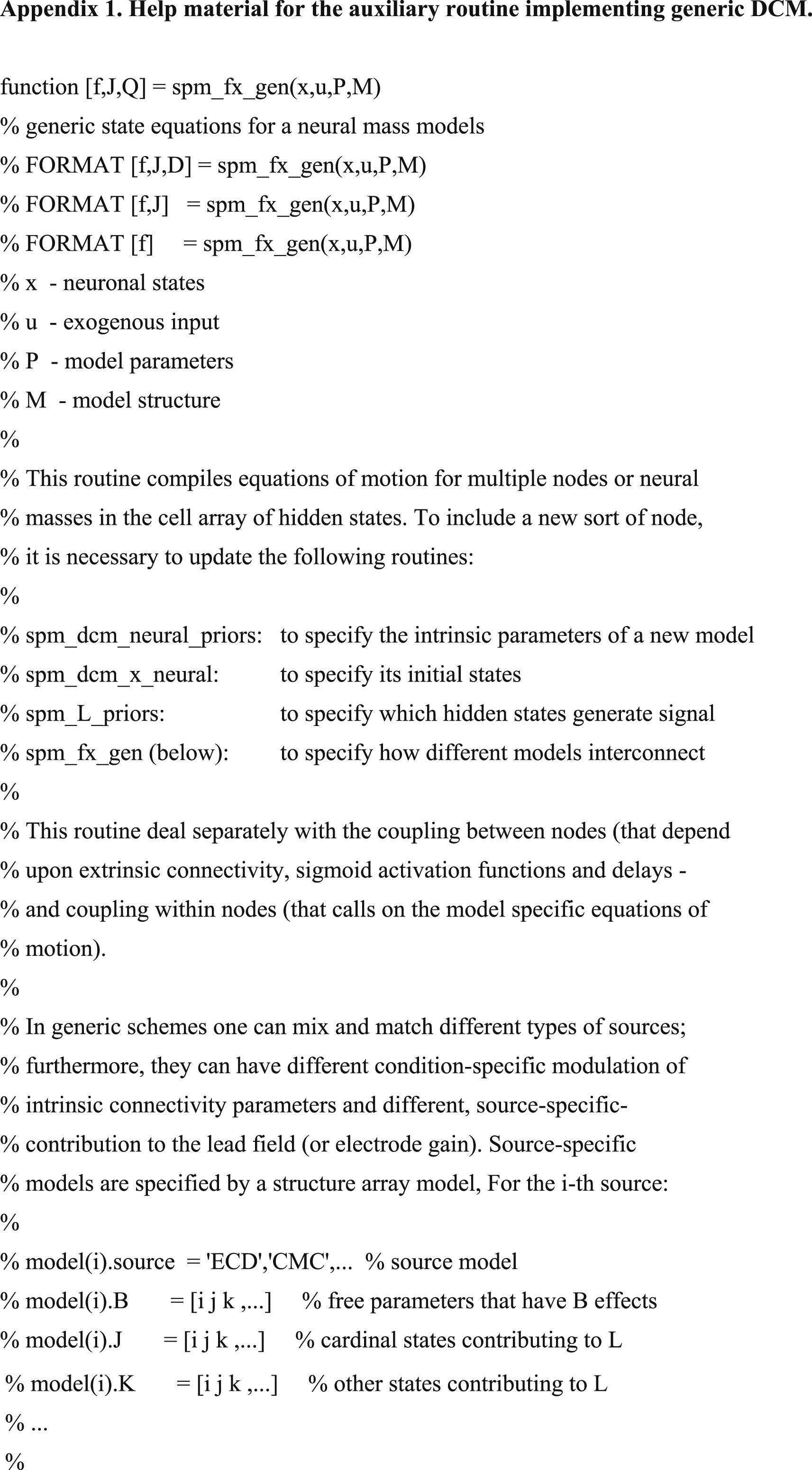

Supplement: Appendix 1 [file EMS86719-supplement-Appendix_1.jpg]
